# The effect of khat consumption on work performance in small businesses in the city-center market, Hargeisa, Somaliland

**DOI:** 10.7717/peerj.21430

**Published:** 2026-06-25

**Authors:** Abdulaziz Bashir Abdi, Abdiwahab Hashi, Abdishakur Mohamed Hussein, Abdalgani Aid Almi

**Affiliations:** 1 Research and Data Analysis, Beder University, Hargeisa, Maroodi-Jeeh, Somalia; 2 Institute of Health, Jigjiga University, Jigjiga, Somali Region, Ethiopia; 3 Dean of Economics and Business, Beder University, Hargeisa, Maroodi-Jeeh, Somalia; 4 Department of Research and Innovation, Beder University, Hargeisa, Maroodi-Jeeh, Somalia

**Keywords:** Khat, Qat, Drugs, Chewing, Consumption, Psychoactive, Cathinones, Cathine, Time, Green

## Abstract

**Background:**

Khat use is an increasing public health concern in Somaliland, linked to various mental, physical, social, and psychological problems.

**Objective:**

This study examined the effect of khat consumption on work performance among small business employees in the City-Center Market, Hargeisa, Somaliland.

**Methodology:**

A quantitative cross-sectional design was employed, with purposive sampling of 150 male respondents. Data were collected through a structured Likert-scale questionnaire measuring khat consumption patterns and work performance. Reliability was confirmed using Cronbach’s alpha, and multiple linear regression analysis was applied to assess the predictive effect of khat consumption on work performance. Demographic and occupational variables (education level, marital status, employment type, type of khat commonly chewed, and age categories) were included as controls.

**Results:**

Regression analysis showed khat consumption was significantly associated with work performance (B = 0.774, *β* = 0.879, *t* = 15.062, *p* < 0.001). Higher levels of khat use were associated with lower work performance, while other variables including education level (*p* = 0.281), marital status (*p* = 0.548), employment type (*p* = 0.902), type of khat (*p* = 0.700), and age (*p* = 0.429) showed no statistically significant effects.

**Conclusion:**

The study suggests that khat consumption may negatively influence employee productivity, while demographic and occupational factors appear to have limited influence. These findings highlight the importance of workplace awareness and policy measures aimed at reducing the potential adverse impact of khat use on work performance.

## Introduction

Khat (*Catha edulis* Forssk, Celastraceae) is a shrub or tree growing 1–25 m tall, widely cultivated in East Africa, from Ethiopia to Madagascar, and extending to Yemen and parts of Saudi Arabia across the Red Sea. It is known by various names, including Chat (Amharic), Gat, Khat, Qat (Arabic), Miraa, Mlonge (Swahili), and Bushman’s tea (South Africa) (Al-Hebshi & Skaug, 2005). Historically, khat leaves have been chewed for centuries in the Eastern part of Africa and the Arabian Peninsula for their stimulant effects. The plant contains cathinone (Schedule I) and cathine (Schedule IV), psychoactive compounds with amphetamine-like properties. Habitual khat use has been linked to adverse health outcomes, including increased blood pressure, cardiovascular complications, and psychiatric issues such as psychosis, depression, and aggressive behavior, and may also contribute to economic and social challenges (Balint, Falkay & Balint, 2009).

The prevalence of khat use has increased globally, in part due to migration to Europe and North (Al-Duais & Al-Awthan, 2021). Cathinone, the primary psychoactive compound, is also classified as a new psychoactive substance in synthetic forms, with cellular and molecular effects contributing to various health risks (Abou-Elhamd et al., 2021; Patel, 2019). In Nairobi, for example, up to 80% of a high-risk population reported concurrent use of cannabis, tobacco, or other substances alongside khat (Ongeri et al., 2023). Similarly, in Somalia, high-frequency khat chewing among participants was reported to occur daily or multiple times per week, typically during the afternoon and evening hours (Awale & Sheikh Ali, 2018).

Frequent khat use has been associated with psychological dependence, withdrawal symptoms, and functional impairments (Silva et al., 2022; Young et al., 2016). A systematic review highlighted that escalating or problematic use can lead to cardiovascular and neurobehavioral complications (Mihretu et al., 2019). Regionally, frequent consumption has been linked to increased blood pressure in Ethiopia (Woldeamanuel & Geta, 2019) and oral health disorders in Djibouti (Bennani & Mohamed, 2021). Beyond health outcomes, habitual use has social and economic consequences, including decreased marital satisfaction (Maalim, 2018), reduced work hours, poor time management, and negative effects on household economic stability (Omar, Kennedy & Mbugua, 2019).

While most effects are negative, some studies suggest potential short-term benefits of moderate use. Khat may temporarily increase energy, alertness, and motivation, and in certain contexts, moderate use has been associated with improved task efficiency and confidence (Gudata, Cochrane & Imana, 2019; Wood et al., 2024a). Communities sometimes perceive longer chewing sessions as a way to sustain energy during extended work activities (Wood et al., 2024b). Nevertheless, prolonged use carries serious risks, including cardiovascular disease (Amer, Abdalla & Ayad, 2020) and neurobehavioral impairments (Ahmed et al., 2021). The dose-dependent effects remain unclear, with some studies reporting inconsistent links between consumption levels and work performance (Olani et al., 2023).

Khat consumption spans diverse societal groups. Both uneducated individuals and professionals in Somaliland have been observed chewing khat, sometimes as a means of political expression or social credibility (Hansen, 2013). The practice may reinforce clan identities and encourage passivity, illustrating broader sociopolitical implications. Among university staff, khat use has been associated with absenteeism, tardiness, and reduced motivation (Gelaw & Haile-Amlak, 2004). These findings highlight the complex physiological, cognitive, and behavioral consequences of khat use and suggest potential implications for workplace productivity.

Khat has significant negative effects on individuals across multiple aspects of life. As a potent psychoactive substance, it is associated with numerous health problems, economic challenges, financial strain, and, in some contexts, political instability. Although extensive research has examined the health and social consequences of khat consumption, relatively little attention has been given to its potential impact on work performance. While khat chewing can consume considerable time, it remains unclear whether this practice directly affects workplace productivity. To the best of our knowledge, no previous studies have specifically explored this relationship.

Consequently, this study aimed to investigate the impact of khat chewing on work performance, with a particular focus on men. It examined the association between khat consumption and work performance, providing insights into its potential effects on individuals’ productivity, morale, and emotional well-being. The research focused on small businesses in the city-center market of Hargeisa, Somaliland, to determine whether a correlation exists between khat consumption and work performance.

## Methods and Materials

### Aim and study design

This study employed a quantitative, cross-sectional design to examine the relationship between khat consumption and work performance among male small-business owners in the city center of Hargeisa, Somaliland. The objective was to quantify the impact of khat use on measurable indicators of work performance, including time management and productivity.

### Study setting

The study was conducted in Hargeisa, the capital and largest city of Somaliland, which had an estimated population of approximately 1.2 million in 2021. Data were collected at the central market, selected for its accessibility to khat consumers. Fieldwork was conducted over a 15-day period (October 5–20, 2025) in quiet, private locations to ensure participant comfort and confidentiality.

### Sampling and data collection

A purposive sampling strategy was employed to recruit participants who met the inclusion criteria. Respondents were first asked whether they chewed khat; only those who reported chewing were included, resulting in a final sample of 150 male participants. The study focused on males because, within the local cultural context, khat consumption is predominantly practiced by men. Women who chew khat typically do so privately due to social stigma, which makes recruiting a sufficient female sample challenging.

Participants were approached in person, informed about the purpose of the study, and voluntarily completed a questionnaire. Trained research assistants familiar with the local context facilitated the data collection process. Completed questionnaires were securely stored and subsequently entered into a database for analysis.

### Sample size and power

A *post-hoc* power analysis was conducted to assess the adequacy of the sample size (*N* = 150) for multiple linear regression with nine predictors, including khat consumption as the primary independent variable and age, education, daily income, marital status, employment type, work experience, type of khat commonly chewed khat as controls. Assuming a medium effect size (*f*^2^ = 0.15) and a significance level of 0.05, the calculated power exceeded 0.99, indicating that the study was sufficiently powered to detect medium to large effects on work performance.

### Measures and instrumentation

#### Independent variable: khat consumption

Khat consumption was operationalized as the habitual chewing of *Catha edulis* leaves. It was measured using a structured questionnaire assessing three dimensions: frequency (days per week), quantity (bundles per session), and duration (years of use) (Al-Motarreb et al., 2005). Items for khat consumption were adapted from the Problematic Khat Use Screening Test (PKUST-17) and related studies to ensure reliability and contextual relevance (Mihretu et al., 2022). The final khat consumption scale consisted of six items, with a Cronbach’s alpha of 0.686, indicating acceptable internal consistency.

#### Dependent variable: work performance

Work performance was operationalized as an individual’s efficiency and effectiveness in completing job-related tasks. It was assessed using a six-item scale covering productivity, punctuality, time management, and task completion, rated on a 5-point Likert scale (higher scores indicate better performance) (Campbell et al., 1993). Items were adapted from previous interview guides and work culture assessments to maintain validity (Gudata, Cochrane & Imana, 2019; Tesfa et al., 2025). Reliability testing yielded a Cronbach’s alpha of 0.739, indicating acceptable internal consistency.

The final questionnaire contained 12 items covering khat use patterns and perceived effects on work performance. The instrument was designed to be culturally relevant and psychometrically sound, ensuring reliable measurement of both variables.

### Statistical methods

Descriptive statistics, including means and standard deviations, were used to summarize khat consumption and work performance variables. A multiple linear regression model was estimated to examine the association between khat consumption and overall work performance. Overall work performance served as the dependent variable. Khat consumption was entered as the main independent variable, while education level, marital status, employment type, type of khat commonly chewed, and age categories were included as control predictors. All predictors were entered simultaneously using the Enter method. Results are reported as unstandardized coefficients (B), standardized coefficients (*β*), *t*-values, and *p*-values. Collinearity diagnostics (Tolerance and Variance Inflation Factor (VIF)) were examined to assess multicollinearity. All analyses were conducted using SPSS version 27.

### Quality control and ethical considerations

Ethical approval was obtained from the Beder University Institutional Review Board (IRB) (Approval No: Rct BIU/AC&QA/483/25). Participants were approached in person and verbally informed about the study’s purpose, procedures, and the voluntary nature of participation. They were assured of confidentiality and reminded that they could withdraw at any time without consequence. Verbal informed consent was obtained from all participants prior to data collection, as approved by the IRB.

All research assistants received training in ethical conduct and data collection protocols. Regular supervision and quality checks were conducted throughout data collection to ensure adherence to ethical standards and improve reliability (Cruz et al., 2009).

## Results and Discussions

Table 1 presents the socio-demographic characteristics of the 150 male respondents. Most participants were between 25–31 years (32.0%) and 32–38 years (30.7%), representing a predominantly young and middle-aged group. The majority had no formal education (62.7%), followed by primary (20.7%) and secondary education (12.7%), while only 4.0% had university education. Nearly half were single (47.3%), 29.3% were married, and smaller proportions were divorced (13.3%), separated (8.0%), or widowed (2.0%). Most participants were employed full-time (94%) and had over seven years of work experience (59.3%), indicating stable employment. Regarding khat type, Jebis was most commonly used (82.7%), followed by Mac (10.0%), Dadar (4.7%), and Miraa (2.7%).

**Table 1:** Socio-demographic characteristics of 150 male small business employees in Hargeisa, Somaliland.

**SN**	**Categories**	**Sub-categories**	**Frequency (n)**	**Valid percentage (%)**
1	Gender	Male	150	100.0%
		Female	0	0.0%
2	Age Groups	Group 1 (18–24)	44	29.3%
		Group 2 (25–31)	48	32.0%
		Group 3 (32–38)	46	30.7%
		Group 4 (39–45)	11	7.3%
		Group 5 (46–52)	1	0.7%
		Group 6 (53+)	0	0.0%
3	Education Level	None	94	62.7%
		Primary	31	20.7%
		Secondary	19	12.7%
		University	6	4.0%
4	Marital Status	Single	71	47.3%
		Married	44	29.3%
		Divorced	20	13.3%
		Separated	12	8.0%
		Widowed	3	2.0%
5	Employment Type	Part-time	9	8.7%
		Full-time	141	91.3%
6	Work Experience	1–2 years	7	4.7%
		3–4 years	21	14.0%
		5–6 years	33	22.0%
		more than 7 years	89	59.3%
7	Type of Khat	Miraa	4	2.7%
		Mac	15	10.0%
		Dadar	7	4.7%
		Jebis	124	82.7%

Participants reported high levels of khat use across all measured dimensions (see Table 2). Frequency, daily chewing duration, irritability when unable to chew, financial expenditure, loss of control, and chewing during work hours all showed high mean scores (M range: 4.827–4.907, SD range: 0.354–0.488), reflecting the prevalence and intensity of khat use in this population.

**Table 2:** Descriptive statistics of khat consumption.

**No.**	**Item**	**Response scale/** ** Anchors**	**N**	**Mean**	**Std. deviation**
1	During the past three months, how often have you chewed khat?	1 = Never, 2 = Rarely, 3 = Sometimes, 4 = Often, 5 = Very often	150	4.9133	.34635
2	During the past three months, how much time (in hours) do you spend chewing khat per day?	1 = Less than 1 h, 2 = 1–2 h, 3 = 3–4 h, 4 = 5–6 h, 5 = 7 or more hours	150	4.8933	.45090
3	During the past three months, how often have you experienced irritability when you didn’t chew khat?	1 = Never, 2 = Rarely, 3 = Sometimes, 4 = Often, 5 = Very often	150	4.9000	.36176
4	During the past three months, to what extent have you spent money on khat that you needed for other things?	1 = Not at all, 2 = A little, 3 = Somewhat, 4 = A lot, 5 = Extremely	150	4.8267	.44491
5	During the past three months, to what extent have you felt unable to control your khat chewing?	1 = Not at all, 2 = A little, 3 = Somewhat, 4 = A lot, 5 = Extremely	150	4.9067	.35420
6	During the past three months, how often have you chewed khat before or during work hours?	1 = Never, 2 = Rarely, 3 = Sometimes, 4 = Often, 5 = Very often	150	4.9000	.48811
	Overall Mean:			**4.89**	

Work performance indicators similarly reflected substantial impairment associated with khat use (see Table 3). Participants frequently reported failing to meet work expectations, arriving late, leaving early, feeling tired or less productive, and difficulty concentrating. Complaints from colleagues or customers were also common. Overall, the mean scores (M range: 4.827–4.940, SD range: 0.289–0.454) highlight consistently high levels of work performance issues among respondents.

**Table 3:** Descriptive statistics of work performance (*N* = 150).

**No.**	**Items**	**Response scale**	**N**	**Mean**	**SD**
1	During the past three months, how often have you failed to do what was normally expected of you at work because of khat use?	1 = Never, 2 = Rarely, 3 = Sometimes, 4 = Often, 5 = Very Often	150	4.9133	.34635
2	During the past three months, how often have you arrived late to work due to khat chewing?	1 = Never, 2 = Rarely, 3 = Sometimes, 4 = Often, 5 = Very Often	150	4.9400	.28918
3	During the past three months, how often have you left work early to chew khat?	1 = Never, 2 = Rarely, 3 = Sometimes, 4 = Often, 5 = Very Often	150	4.9067	.35420
4	During the past three months, how often have you felt tired or less productive at work after chewing khat?	1 = Never, 2 = Rarely, 3 = Sometimes, 4 = Often, 5 = Very Often	150	4.8267	.44491
5	During the past three months, how often have you had difficulty concentrating on your work after chewing khat?	1 = Never, 2 = Rarely, 3 = Sometimes, 4 = Often, 5 = Very Often	150	4.9067	.35420
6	During the past three months, how often have you received complaints from customers or colleagues about your work performance after chewing khat?	1 = Never, 2 = Rarely, 3 = Sometimes, 4 = Often, 5 = Very Often	150	4.9067	.35420
	**Overall Mean:**			**4.900**	

Table 4 presents the results of the multiple regression analysis examining predictors of work performance. Khat consumption was the only statistically significant predictor (*B* = 0.774, *β* = 0.879, *t* = 15.062, *p* < 0.001), indicating that higher levels of khat use were associated with poorer work performance. Other variables, including education level, marital status, employment type, type of khat chewed, and age categories, did not significantly predict work performance (*p* > 0.05). These results suggest that khat consumption is the dominant factor influencing work performance, whereas demographic and occupational characteristics contribute minimally.

**Table 4:** Regression analysis predicting work performance.^a^

**Predictor**	**B (Unstandardized)**	**Std. error**	**Beta (Standardized)**	*t*	*p*
Constant	1.142	0.278	–	4.102	<0.001
Khat Consumption	0.774	0.051	0.879	15.062	<0.001
Education Level	−0.014	0.013	−0.063	−1.087	0.281
Marital Status	0.008	0.013	0.037	0.604	0.548
Employment Type	−0.005	0.039	−0.008	−0.123	0.902
Type of Khat Commonly Chewed	0.006	0.016	0.025	0.387	0.700
Age Categories	−0.009	0.011	−0.046	−0.796	0.429

**Notes.**

a, Dependent variable: work performance.

As shown in Table 5, the model explained a substantial portion of the variance in work performance (*R*^2^ = 0.794, Adjusted *R*^2^ = 0.774), indicating that approximately 77.4% of the variation in work performance can be attributed to the combined effects of age categories, type of khat commonly chewed, khat consumption, education level, employment type, and marital status. The high *R* value (*R* = 0.891) suggests a strong correlation between the predictors and the dependent variable.

**Table 5:** Model summary of the regression analysis predicting work performance.

**Model**	**R**	**R square**	**Adjusted R square**	**Std. error of the estimate**
1	0.891^a^	0.794	0.774	0.08985

**Notes.**

a Predictors: (Constant), Age Categories, Type of Khat Commonly Chewed, Khat Consumption, Education Level, Employment Type, Marital Status.

The analysis of variance (ANOVA) results (Table 6) showed that the overall regression model was statistically significant (F (6, 65) = 41.642, *p* < 0.001), confirming that the set of predictors reliably explains variations in work performance among respondents.

**Table 6:** ANOVA summary for the regression model predicting work performance.^a^

**Model**	**Sum of squares**	**df**	**Mean square**	**F**	**Sig.**
Regression	2.017	6	0.336	41.642	<0.001^b^
Residual	0.525	65	0.008		
Total	2.542	71			

**Notes.**

a Dependent Variable: Work Performance.

b Predictors: (Constant), Age Categories, Type of Khat Commonly Chewed, Khat Consumption, Education Level, Employment Type, Marital Status.

## Discussion

The present study examined the association between khat consumption and work performance among small business employees in Hargeisa while controlling for demographic and occupational characteristics.

The findings showed that khat consumption was a statistically significant predictor of work performance, with higher levels of use associated with poorer workplace functioning.

This pattern is consistent with a growing body of research linking habitual khat consumption to reduced productivity, absenteeism, impaired concentration, and occupational inefficiency across different contexts (Ali, Mughal & Mawusi, 2021; Awadalla & Suwaydi, 2017; Gelaw & Haile-Amlak, 2004).

Rather than being confined to specific occupational groups, the broader literature suggests that sustained khat use may be associated with functional impairment in diverse working populations (Cox & Rampes, 2003; Hoffman & Al’Absi, 2010).

Although cathinone, the primary psychoactive component of khat, may produce short-term increases in alertness and mood, these acute effects appear to be followed by fatigue, irritability, sleep disturbance, and cognitive decline, thereby undermining sustained work performance over (Cox & Rampes, 2003; Hoffman & Al’Absi, 2010; Kline, 1996).

Accordingly, the present findings align with the dominant interpretation in the literature that any perceived short-term productivity enhancement is likely outweighed by longer-term occupational impairment (Gebissa, 2010; Hassan, Gunaid & Murray-Lyon, 2007).

However, the strength of the standardized beta coefficient and explained variance should be interpreted with caution, as they may be influenced by methodological constraints, particularly restricted variability in the data, suggesting a potential ceiling effect. Limited variation in both khat consumption and work performance may have inflated or distorted the observed relationships. Therefore, while the results indicate an association between khat use and work performance, the true magnitude of this relationship should be interpreted as provisional (Podsakoff et al., 2003).

Therefore, while the results provide preliminary evidence of an association between khat use and work performance among male market workers in Hargeisa, the true magnitude of this relationship cannot be definitively inferred from the present cross-sectional data (Ali, Mughal & Mawusi, 2021).

Similarly, the absence of statistically significant effects for education, marital status, employment type, khat type, and age should not be interpreted as conclusive evidence that these variables lack influence, as model specification and measurement constraints may attenuate or distort their effects (Gelman & Hill, 2007).

Existing research indicates that sociodemographic factors often shape patterns of khat consumption rather than directly determining occupational outcomes, suggesting that their effects may operate indirectly through behavioral pathways (Addis et al., 2019).

Once consumption behavior is accounted for, its functional consequences may manifest relatively uniformly across demographic groups, which may help explain the non-significant findings observed in the present analysis (Awadalla & Suwaydi, 2017).

Measurement instruments should be developed in accordance with international psychometric standards, including item selection based on moderate item difficulty and high corrected item–total correlations to ensure adequate discrimination and scale coherence (DeVellis & Thorpe, 2021; Nunnally & Bernstein, 1994).

At the same time, reliability coefficients should reach acceptable thresholds while preserving adequate construct breadth, as excessive homogeneity may narrow the conceptual coverage of the measure (Cronbach, 1951; Schmitt, 1996). The well-established validity-reliability trade-off suggests that extremely high internal consistency can indicate that an instrument captures only a limited facet of the construct rather than its full theoretical domain, thereby potentially constraining its external validity (Schmitt, 1996).

Future instruments measuring khat consumption should differentiate between frequency, quantity, dependency symptoms, and functional impairment in order to capture its multidimensional nature (Humeniuk et al., 2016).

Similarly, work performance measures should incorporate multiple dimensions such as task performance, contextual performance, and counterproductive work behaviors to enhance construct validity and reduce mono-method bias (Borman & Motowidlo, 1997).

Longitudinal designs would further clarify whether the association observed reflects temporary stimulant effects, cumulative impairment, or bidirectional dynamics between work stress and substance use (Hoffman & Al’Absi, 2010).

Overall, the present study offers preliminary evidence suggesting an association between khat consumption and reduced work performance in a Somaliland market context, yet the strength of this association should be regarded as provisional pending replication with more robust measurement strategies.

## Limitations

This study has several limitations that should be considered when interpreting the findings.

First, a major limitation of this study is the presence of restricted variability (ceiling effect) in the measured variables. Responses for both khat consumption and work performance were highly concentrated at the upper end of the scale, resulting in limited variance. This restricted range may reduce statistical sensitivity and affect the accuracy of estimated relationships between variables. Future research should employ more discriminative measurement tools and include both khat users and non-users to improve variability and enhance analytical precision.

Second, the sample was restricted to male business owners working in Hargeisa market, thereby excluding female participants and other occupational groups. This restricted sampling frame limits the generalizability of the findings to the broader population of khat users in Somaliland. Future studies should include both men and women and recruit participants from diverse employment sectors to enhance external validity.

Third, the use of purposive sampling, although appropriate for exploratory research, may introduce selection bias and reduce representativeness. Employing probability-based sampling strategies with larger and more heterogeneous samples would strengthen the generalizability of future research findings.

Fourth, both khat consumption and work performance were measured using self-report instruments administered at a single time point, which may introduce common method variance. However, this is not considered the primary source of bias in this study. Compared to other limitations, particularly the restricted variability (ceiling effect) observed in the data, the influence of common method variance is likely to be less substantial. Future research should incorporate multi-source assessments, such as supervisor ratings or objective productivity indicators, to obtain more robust estimates.

Fifth, although verbal informed consent was obtained and approved by the University’s ethics committee, written consent was not collected, which may limit documentation of ethical procedures. Subsequent studies should incorporate written informed consent to strengthen procedural transparency and ethical rigor.

Finally, the cross-sectional design precludes causal inference, as temporal ordering between khat consumption and work performance cannot be established. Longitudinal or experimental designs are needed to clarify causal pathways and examine potential bidirectional dynamics between substance use and occupational functioning.

## Conclusion

This study identified a negative association between khat consumption and work performance among male small business employees in Hargeisa. Higher levels of khat use were associated with lower reported productivity, while demographic and occupational factors showed no statistically significant relationships with work performance. However, these findings should be interpreted with caution due to methodological limitations, particularly a potential ceiling effect resulting from restricted variability in the measured variables.

## Recommendations

Building on these findings, the following recommendations are proposed:

### Awareness and education programs

Programs that provide education on the health, social, and economic consequences of khat use should be introduced to help business owners and employees make more informed decisions about consumption habits.

### Workplace policies and support

Employers and organizations should establish workplace policies that discourage khat use during working hours and promote productivity-enhancing alternatives, such as health counseling or recreational programs.

### Further research and policy development

Policymakers and researchers should collaborate to conduct longitudinal studies examining the long-term effects of khat consumption on work performance, guiding evidence-based interventions and national policy formulation.

## Supplemental Information

10.7717/peerj.21430/supp-1 Supplemental Information 1 Questionnaire of the Khat consumption on work performance Dataset containing the original survey responses and variables used in the analysis of the relationship between khat consumption and work performance.

## Additional Information and Declarations

### Data Availability

The following information was supplied regarding data availability: The raw data are available in the Supplemental Files.
